# No transcriptional evidence for active Na_v_ channels in two classes of cancer cell

**DOI:** 10.1080/19336950.2019.1644858

**Published:** 2019-07-22

**Authors:** Supanida Hompoonsup, David Chambers, Patrick Doherty, Gareth Williams

**Affiliations:** a Wolfson Centre for Age-Related Diseases, King’s College London, London, UK; b Learning Institute, King Mongkut’s University of Technology Thonburi, Bangkok, Thailand

**Keywords:** Ion channel, voltage-gated sodium channel, Nav1.7, tetrodotoxin, ProTx-II, gene expression profiling, microarray

## Abstract

Voltage-gated sodium channel (Na_v_) expression in non-excitable cells has raised questions regarding their non-canonical roles. Interestingly, a growing body of evidence also points towards the prevalence of aberrant Na_v_ expression in malignant tumors, potentially opening a new therapeutic window. In this study, the transcriptional consequences of channel inhibition were investigated in non-small cell lung carcinoma H460 and neuroblastoma SH-SYSY cell lines, that both express Na_v_1.7. Channel activity was blocked by the application of both selective, ProTx-II, and non-selective, tetrodotoxin, inhibitors. Global gene expression profiling did not point to any statistically significant inhibition-associated perturbation of the transcriptome. A small subset of genes that showed relatively consistent changes across multiple treatments were further assayed in the context of a multiplex bead expression array which failed to recapitulate the changes seen in the global array. We conclude that there is no robust transcriptional signature associated with the inhibition of two sodium channel expressing cancer cell lines and consequently sodium channel inhibition will not lend itself to therapeutic approaches such as transcription-based drug repurposing.

## Introduction

Voltage-gated sodium (Na_v_) channels were originally characterized as prominent players in signal conduction in excitable cells, such as neurons and myocytes, principally through the regulation of cellular ion balance [1,2]. Subsequent findings led to speculation that Na_v_ channels may also be involved in a wider repertoire of cellular processes [3]. One notable observation was that Na_v_ channels are not exclusively present in excitable cells and appear to take on roles that are not necessarily related to action potential generation and cellular excitability [4]. For example, functional expression of the channels has been observed in cancer cells, with recordings of voltage-gated sodium currents in human leukemia [5] and small-cell lung cancer cells [6]. Over the years, innumerable studies from independent research groups have added to a rapidly expanding body of evidence for the functional expression of Na_v_ channels in non-excitable cells of wide-ranging backgrounds including, red blood cells [7], macrophages [8], oligodendrocytes [9], dendritic cells [10], and fibroblasts [11]. The expression of Na_v_ channels in these cells is dynamic, and may alter depending on the developmental, physiological, and pathological state [12,13]. This dynamic expression profile has been linked to distinct biological states of the cell serving to implement diverse biological functions, e.g. motility, endosomal acidification, and phagocytosis [14,15].

The functional relevance of Na_v_ channels in a non-excitable context has been most intensely investigated in cancerous cells. This focus could certainly be explained in part by the fact that Na_v_ channels are anomalously expressed in a wide range of tumors, including, lymphoma, breast cancer, melanoma, colon, and ovarian cancer [16]. In some cases, the channels are not present in the corresponding healthy tissues, e.g., breast cancer [17]. On the other hand, in certain cancers, such as glioma and ovarian cancer, Na_v_ channels are expressed in the healthy counterparts [18,19]. In the latter case, the degree of channel overexpression is tied to the aggressiveness of cancer metastasis [20]. The mechanisms by which Na_v_ channels exert their metastasis-related effects are only beginning to be unraveled and thus far results have largely been obtained from breast and prostate cancer studies [21]. Sodium ions are clearly an important factor, still how much of the observed response is due to the fluctuation in Na^+^ concentration, membrane potential, or transporter-assisted ion exchange, has yet to be fully characterized [22]. One school of thought believes the interaction between Na_v_ channels and partner proteins to be critical, based on accumulating evidence that various scaffolding proteins partner with the channels to form multiprotein complexes [23,24].

Na_v_ channels exist in several isoforms which are distributed in distinct cell types and locations, e.g., Na_v_1.1 is expressed in both the CNS and PNS to Na_v_1.9 predominantly in the PNS [25]. Mutations in Na_v_1.7 are associated with painful channelopathies and congenital insensitivity to pain [26,27]; it comes as no surprise that this channel has been a prime target for pain management [28]. Interestingly, parallel work has revealed Na_v_1.7 upregulation in a number of tumors, for example, breast [17], prostate [29], gastric [30], and non-small cell lung cancer [31]. This overexpression is reportedly associated with tumor-related transcriptional changes and is linked to the aggressive phenotypes exhibited by the metastatic cells [14,30,32]. It follows that suppression of Na_v_1.7 expression or activity might improve cancer prognosis. In fact, there is speculation that the use of analgesics that engage Na_v_ channels for cancer surgery may have implications in lessening tumor reoccurrence [33].

In the present work, we have sought to elucidate the functional consequence of sodium channel expression in the cancer context by following changes in gene expression upon channel inhibition. The possibility that there are transcriptional changes associated with channel inhibition could potentially open up new avenue for therapeutic screening and possible drug repurposing [34–38] based on the wealth of available drug-associated transcriptional data [39]. Na_v_1.7 has been shown to be the prominent Na_v_ subtype in the H460 lung cancer and SH-SY5Y neuroblastoma cell lines [31,40] and we decided to investigate channel activity in these two cell lines with a specific Na_v_1.7 antagonist and a more general inhibitor. Specifically, the pufferfish derived neurotoxin tetrodotoxin (TTX) has been shown to block a subset of Na_v_ channels (TTX sensitive Na_v_1.1–1.4 and Na_v_1.6–1.7; Pinto, Derkach [41]), and the tarantula derived neurotoxin ProTx-II exhibits Na_v_1.7 selectivity [42].

## Methods

### Pharmacological treatment and microarray

SH-SY5Y neuroblastoma cell line was kindly provided by Dr Paul Francis’ group (Wolfson CARD, KCL UK), and maintained in DMEM-high glucose medium at 37°C, 5% CO_2_. NCI-H460 non-small cell lung cancer cell line was purchased from ATCC USA and maintained in RPMI 1640-GlutaMAX medium at 37°C, 5% CO_2_. Cells were passaged 2–3 times a week. Complete growth media for both cell lines were supplemented with 10% FBS (PAA Laboratories, UK), 100 U/mL penicillin, and 100 μg/mL streptomycin. All culture media and supplements were purchased from Sigma-Aldrich, UK, unless otherwise stated.

For the experiments, SH-SY5Y and H460 cells were seeded at 100,000 cells per well in a 12-well sterile cell culture plate and incubated for 48 h to reach approximately 80% confluence. Cells were then treated for 6 h with 1 μM TTX (Sigma-Aldrich), 1 or 10 nM ProTx-II (SmarTox); as the IC_50_ of ProTx-II for Na_v_1.7 is approximately 1 nM [43], we decided to work with concentrations in the range 1 to 10 nM. Each treatment was replicated in four culture wells. Following this, culture media were removed, and the cells were lysed in Absolutely RNA Miniprep Kit lysis buffer and β-mercaptoethanol (Agilent Technologies, UK). RNA was then extracted, and quality assessed (RNA Integrity Number ≥8). RNA expression levels were measured on Affymetrix Human Genome U133 plus 2.0 (GPL570) chip following the supplier’s recommended procedure. Resulting expression data were pre-processed with Affymetrix MAS5.0.

### Multiplex assay

A custom-made set of paramagnetic microbeads comprising detection probes for 50 genes (44 chosen based on preliminary analysis and 6 housekeepers) was supplied by Luminex (Thermo Fisher Scientific). Assay components were stored at 2–8 ⁰C or −20 ⁰C according to storage specification.

For one well on a 96-well plate, working bead solution was made up by mixing 9.2 µL nuclease-free water, 6.6 µL lysis buffer, 1 µL blocking reagent, 0.2 µL proteinase K, 0.5 µL capture beads, and 2.5 µL probe set, totaling 20 µL. All reagents were supplied as part of the Plex Assay Kit (QuantiGene, UK). Eighty microliters of cell lysate and 20 µL working bead solution were mixed and added to each well. Three blanks and a positive control were included in every run. The assay plate was incubated for 18 h at 54⁰C, 600 rpm. After overnight hybridization, the plate was secured on a magnetic hand-held platform, and the assay wells were rinsed 3X with 100 µ wash buffer. One hundred microliters of pre-amplifier solution was added and incubated for 1 h at 50⁰C, 600 rpm. The incubation-wash step was repeated with an amplifier, and label probe solution. After 3X washes for label probe solution, 100 µL SAPE working reagent (3 µL SAPE per 1 mL SAPE diluent) was added to each assay well and incubated for 30 min at RT, 600 rpm. The wells were washed 3X with SAPE wash buffer. Lastly, 130 µL of SAPE wash buffer was added and mixed well by agitating at 800 rpm for 3 min. The plate was read immediately on the MAGPIX platform with xPONENT software (Luminex, USA).

### Validation of cell type

The NCBI GEO hosts 145,000 samples on this platform, making it the most popular array chip. The relative expression levels of probes were collected for the GEO data and the given cell types. The ranks were scaled to lie between zero for the highest expression probe to unity for the lowest. The relative rank of each probe was defined as $\frac{r_{0}-r}{r_{0}}$ for $r<r_{0}$and $\frac{r_{0}-r}{1-r_{0}}$ for $r>r_{0}$, where r and $r_{0}$ are the average probe ranks over the given cell type samples and the set of samples deposited on GEO, respectively. Probes were then mapped to genes and in the case of degeneracy, the probe with the largest relative rank mapping to the gene. The gene rank profile was taken to be related to the relative gene expression characterizing the cell type.

### Microarray transcription profiles

Transcription profiles for sodium channel inhibition were based on within plate treatment versus control gene expression levels, where the scaled fold was defined as $2\frac{\langle t\rangle -\langle c\rangle }{\langle t\rangle +\langle c\rangle }$, were 〈t〉 and 〈c〉 are the average treatment and control levels, respectively. In the case of the cell type profiles, the averages were taken over the two cell types. Only folds over 20% passing a t-test threshold of 0.05 were considered. Heat maps for individual gene and sample pairs were generated based on the gene expression level relative to the distribution over all samples, $\frac{x-\langle x\rangle }{\sigma_{x}}$, where *x* is the expression level, the brackets indicate the average and the denominator is the standard deviation.

### Bead array transcription profiles

The median level fluorescence of between 50 and 100 bead reads was taken to be the measure of the corresponding gene’s RNA level. Gene expression levels in each well were normalized against the geometric mean of the total gene set. The normalization scheme was chosen to maximize the correlation of the cell type expression profile with that seen in the microarray data. Expression profiles were defined as for the microarray analysis, see above.

## Results

### Cell-specific nature of the microarray profiles

**Figure 1.**
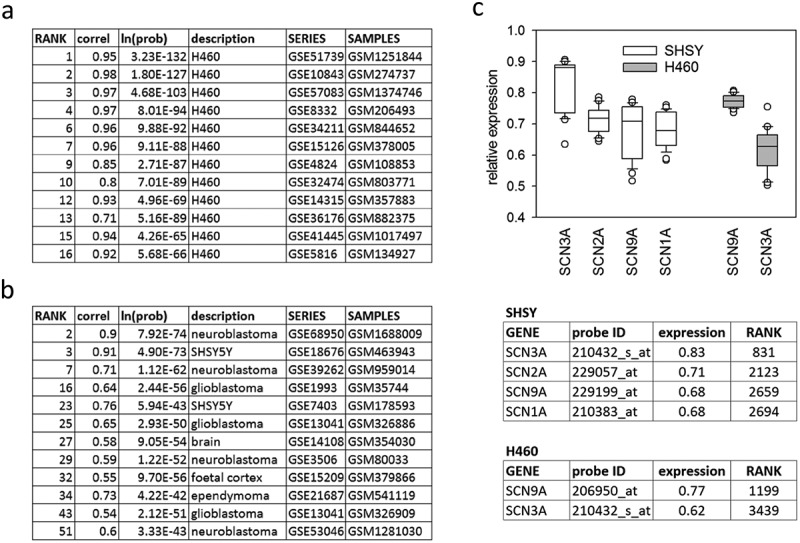
The relative rank expression profile for the H460 (a) and SHSY (b) samples were queried against human samples in SPIED. The top hits are listed in the table, showing that they are enriched by samples from corresponding cell types. The correlation is more pronounced for the H460 cells as these are the subject of more available studies. The SHSY correlations pick out SHSY samples as well as samples from various brain cancer cells. The two cell lines express the sodium channels SCN3A and SCN9A (c). The neuroblastoma cells also express SCN1A and SCN2A.

As a first step in establishing the phenotype of the model cell system, their transcriptional profile was queried against a database of publicly deposited gene expression profiles via SPIED [44,45], see Methods. The top 1000 genes in the H460 and SH-SY5Y or SHSY rank profiles are overwhelmingly up-regulated consisting of (977 up-regulated and 23 down-regulated) for H460 and (950 up-regulated and 50 down-regulated) for SHSY. These profiles served as queries in the SPIED search. It is perhaps worth pointing out here that the level of gene expression unique to a given cell type will tend to be elevated relative to a background consisting of a variety of tissue types. An analogy would be in the context of division of labor one is characterized by what one does not by what one does not do. The top SPIED hits show a high correlation with appropriate cell types, validating the cell lineages, with the H460 query resulting in exclusively H460 profiles, Figure 1(a). The neuroblastoma query results are less exclusively that of the same cell type, but nonetheless picking out 12 profiles of related cell phenotype in the top 51, Figure 1(b).

Expression level of sodium channel types across cell types is shown in Figure 1(c). The lung cancer cells H460 only express SCN3A and SCN9A, whereas the neuroblastoma cells SHSY express SCN1A, SCN2A, SCN3A, and SCN9A.

### Gene expression is not affected by channel inhibition

**Figure 2.**
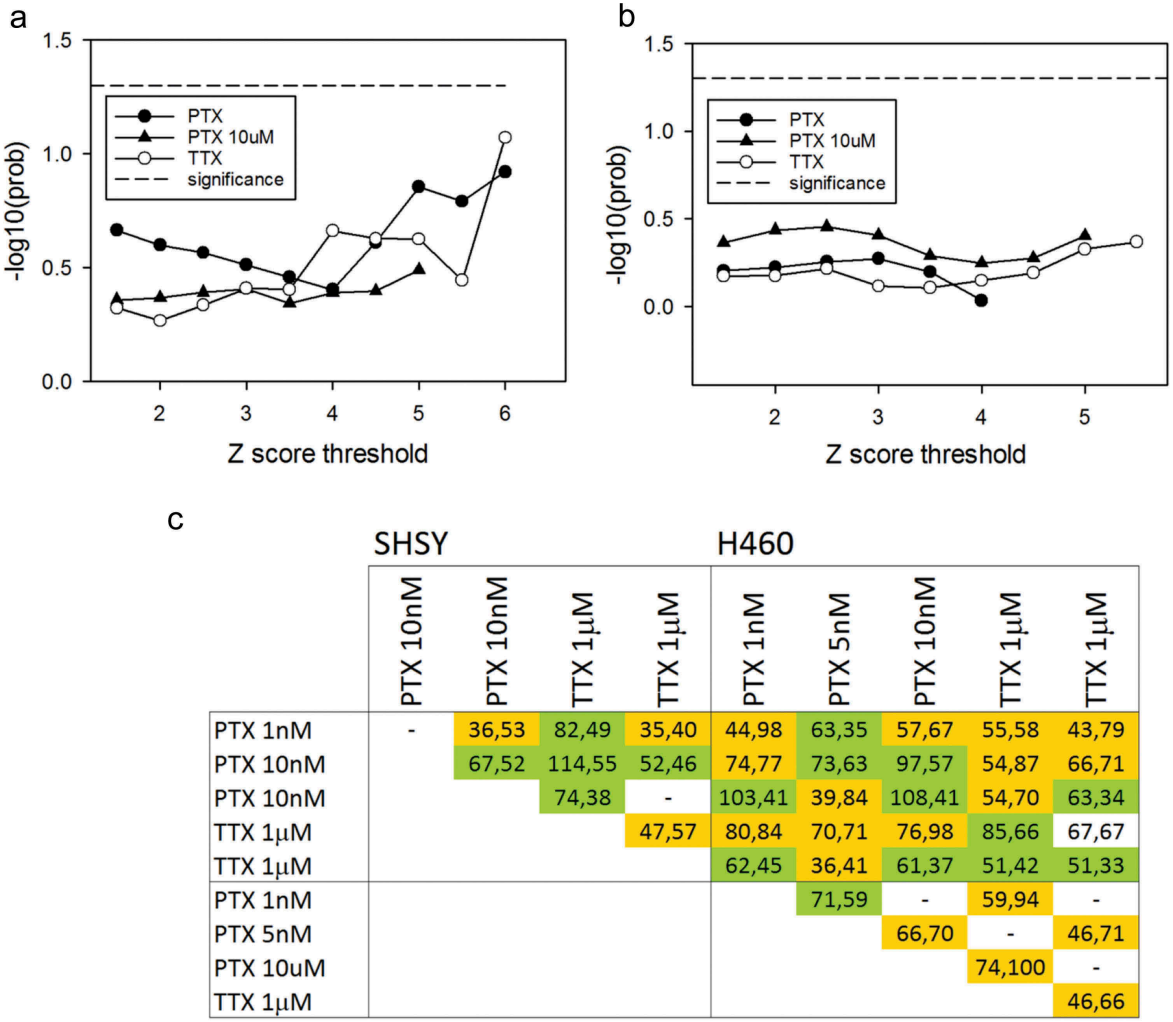
There is no significant transcriptional response upon inhibiting sodium channels in either the lung cancer or neuroblastoma cell lines. Monte Carlo significance test of association of gene expression with treatment type shows that neither the SHSY response (a) or H460 response (b) is significantly elevated beyond a random association of treatment and control groups. The inhibitor expression profiles generated from independent plate replicates show little consistency, see (c). The entries are colored according to the sign of the correlation, green for positive and orange for negative.

To investigate whether the modulation of channel activity has a transcriptional consequence, the extent of gene expression change was determined with samples segregated according to treatment type and compared to a random assignment of sample to treatment type. The extent to which expression is driven by the given treatment is then measurable by a Monte Carlo simulation, counting the number of times a random assignment has a larger perturbed gene set [46]. As can be seen from Figure 2(a,b), neither of the sodium channel inhibitors TTX or PTX pass a statistical threshold of treatment dependent gene perturbation. Individual profiles generated from samples from the various plate series were also compared for consistency in regulation. As can be seen from Figure 2(c) there appears to be little in the way of robustness in the conservation of regulated gene sets, with the numbers going in the same direction alternately surpassing and coming short of those going in opposite directions. It can be concluded that there is no robuts transcriptional signature associated with sodium channel inhibition of the the two cancer cell types, with the caveat that the transcriptional effects would be more pronounced with higher inhibitor concentrations. However, as with all pharmacological studies, one must be wary of non-specificity emerging here, see Park, Carlin [43] and Pinto, Derkach [41]

### Sodium channel gene set

**Figure 3.**
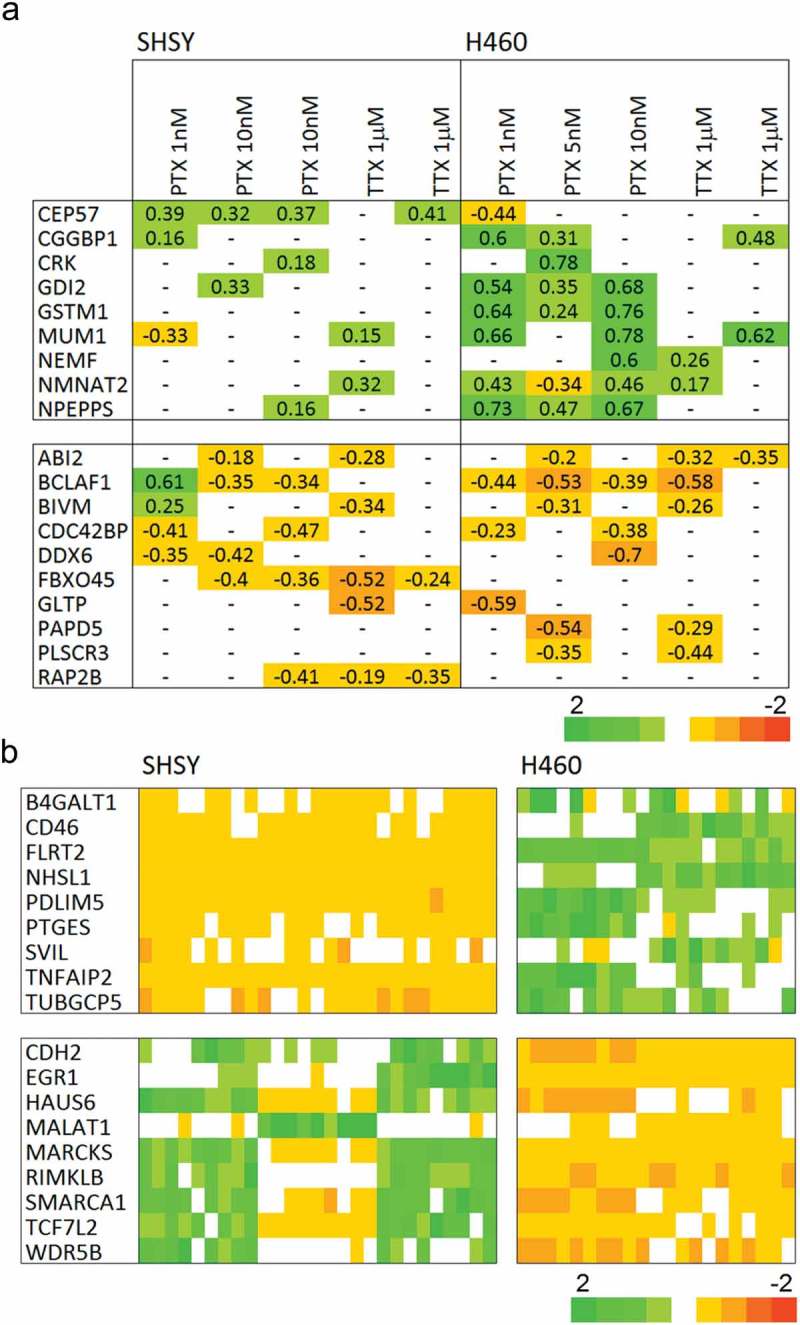
The gene sets for validation. The gene set based on consistently regulated genes by the PTX and TTX inhibitors are shown in (a), the cells are colored according to the scaled fold value, see Methods. Cell type is strongly encoded in relative gene expression (b) and can serve as a positive control for the multiplex array experiments. The cells are colored according to the Z-score of the response relative to the average and standard deviation over the full sample set, $\frac{x-\langle x\rangle }{\sigma_{x}}$, where *x* is the expression level, the brackets indicate the average and the denominator is the standard deviation.

The overall gene expression profiles point to there being little or no effect of channel modulation in either cell type examined. Nonetheless, there may be a restricted gene set that is consistently perturbed by channel modulation. To this end, the separate profiles defined according to cell type, treatment type, and concentration were generated and compared, see Figure 3(a). There is a small population of genes that are consistently perturbed across treatments and it was reasoned that these could facilitate a biomarker for channel modulation. As a positive control for subsequent verification, a gene set was designed based on delimiting the two cell types, see Figure 3(b).

### Bead assay

**Figure 4.**
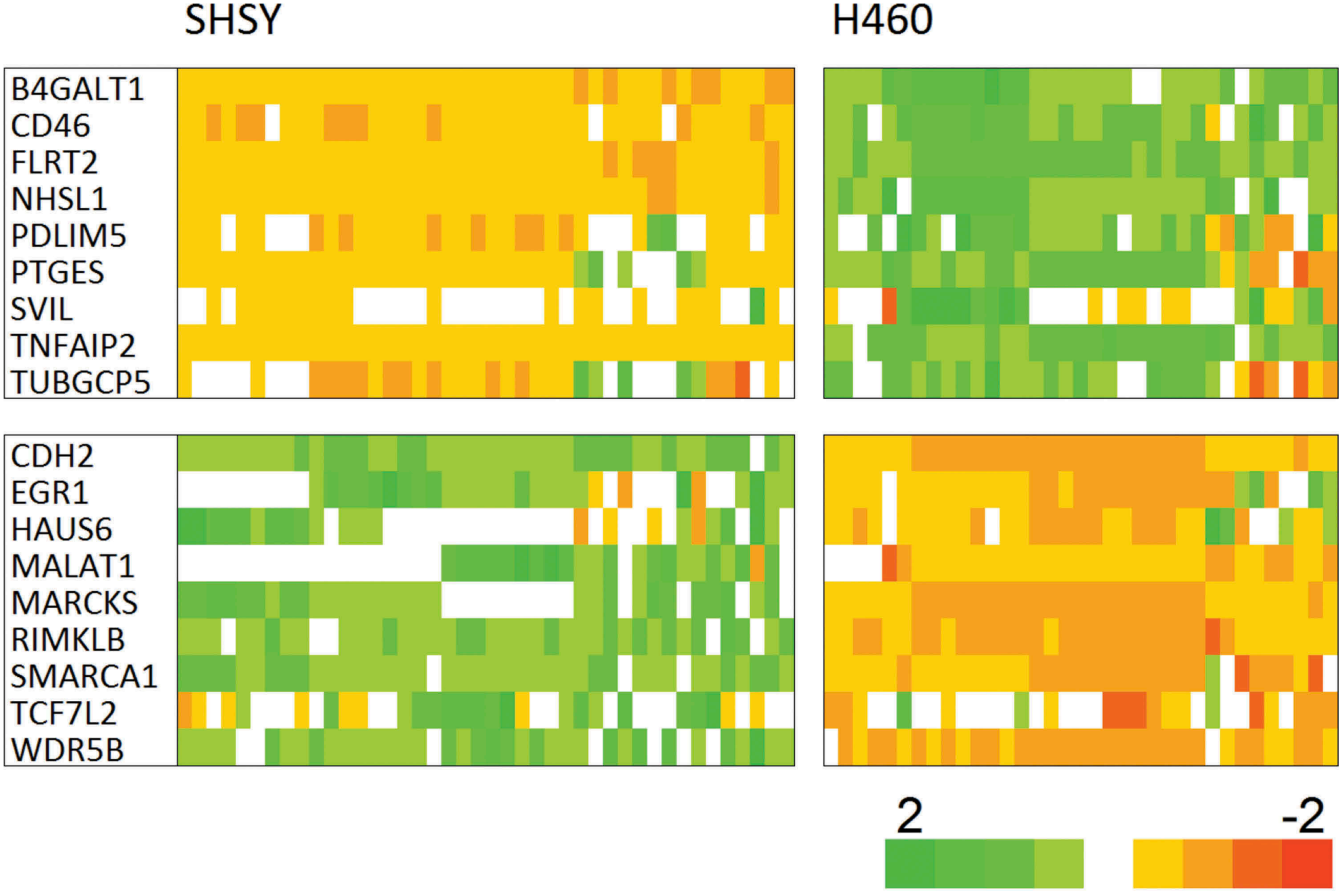
The cell type tracker set effectively segregates the lung cancer and neuroblastoma cells. The cells are colored according to the Z-score of the response relative to the average and standard deviation over the full sample set, $\frac{x-\langle x\rangle }{\sigma_{x}}$, where *x* is the expression level, the brackets indicate the average and the denominator is the standard deviation.

**Figure 5.**
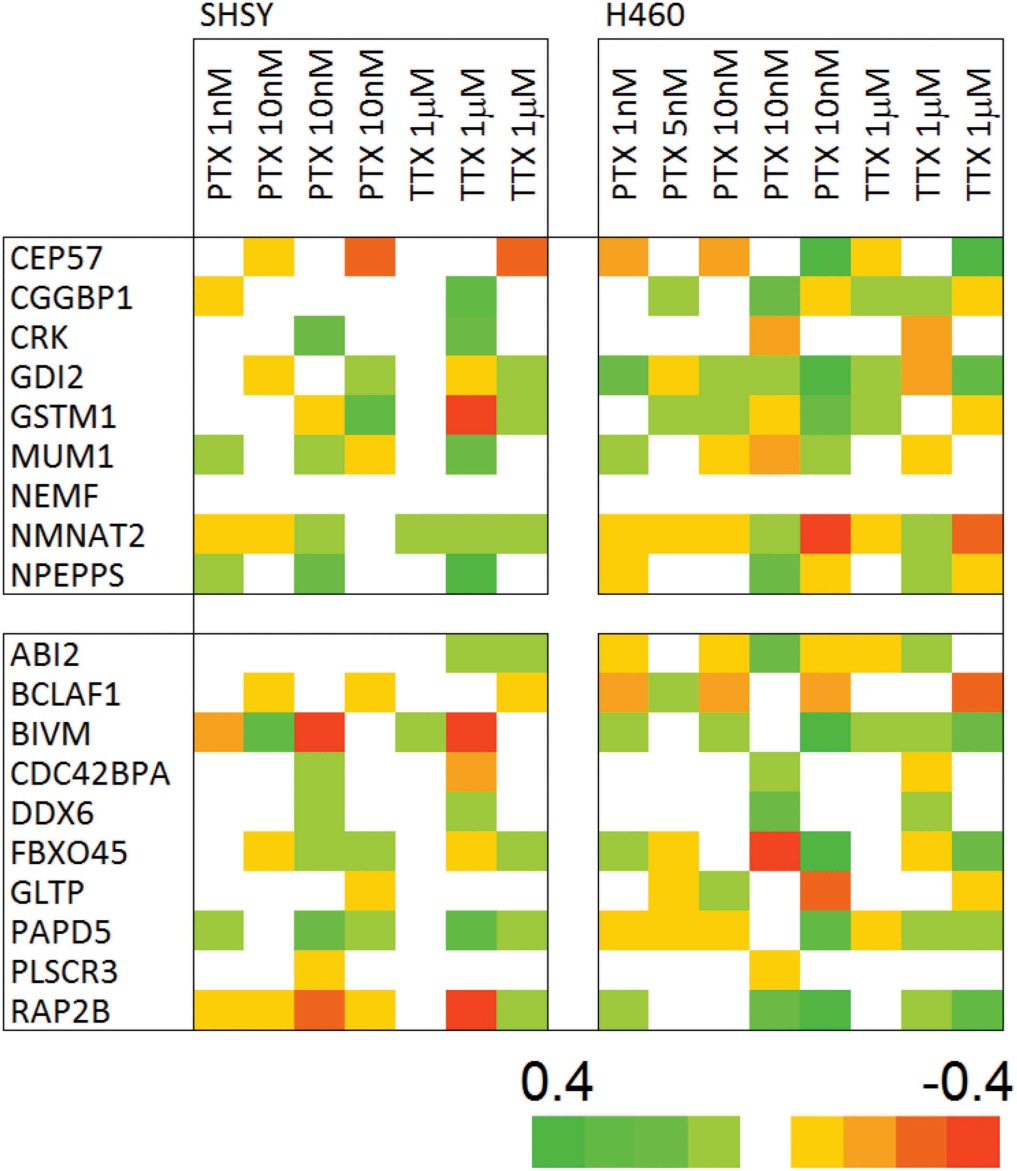
Bead array channel modulator tracker shows no association with sodium channel inhibition. The association of gene expression change with sodium channel inhibition seen in the array experiments, Figure 3(a) is not recapitulated in the bead experiment. The cells are colored according to the scaled fold value, see Methods.

Comparison of cell type-specific expression is in good agreement with the array data, Figure 4 explaining this. The bead assay expression data are given in Figure 5. It is clear that there is no correlation between predicted expression direction change and that observed across 15 independent sodium channel inhibition treatments.

## Conclusions

This study sought to investigate the activity of sodium channels in the context of cancer cells through transcriptional profiling. We first showed that the H460 and SH-SY5Y cell phenotype could be effectively defined through relative gene expression and further showed that a subset of sodium channels is expressed in the two cell types. Having established sodium channel expression, we then determined whether pharmacological intervention would elicit a transcriptional response by applying inhibitor toxins that block Na_v_ channels either non-selectively (TTX) or with Nav1.7 specificity (ProTx-II). Our initial microarray results suggested that these inhibitors do not induce consistent alterations in overall gene expression in either of the two cell lines as indicated by Monte Carlo significance test and inter-profile comparison. Nonetheless, we reasoned that there might still be a restricted gene set that responds to channel modulation and selected a subset of genes from the microarray data for further validation in a multiplex platform. Two distinct multiplex biomarker gene sets were defined to, first of all, validate the platform through cell-specific marker expression and subsequently to test the validity of inhibitor-associated changes observed in the microarray data. The multiplex platform successfully delimited the cell type based on the expression of cell type marker genes but failed to reproduce the putative inhibitor-associated changes. We, therefore, conclude that in the context of gene expression there is no robust sodium channel activity in the two cancer cell lines under normal culture conditions. Consequently, assuming channel inhibition is a potential therapeutic target in cancer our results indicate that transcription-based approaches are unlikely to succeed. However, there is a possibility that the sodium channel activity is transcriptionally silent or that expression changes are not substantial enough for the two independent platforms to detect. For example, by altering membrane potential the channels might cause localized calcium influx and kinase activation in a manner that might impact on migration and/or proliferation in the absence of substantial transcriptional changes. Further, it must be borne in mind that our results are based on cells grown under normal culture conditions and there remains the possibility that the apparently silent channels might be activated by as yet to be identified agents and/or conditions.
